# Glioma Biopsy Based on Hybrid Dual Time-Point FET-PET/MRI—A Proof of Concept Study

**DOI:** 10.3389/fneur.2021.634609

**Published:** 2021-05-11

**Authors:** Jacek Furtak, Józefina Rakowska, Tadeusz Szylberg, Marek Harat, Bogdan Małkowski, Maciej Harat

**Affiliations:** ^1^Department of Neurosurgery, 10th Military Research Hospital, Bydgoszcz, Poland; ^2^Department of Pathomorphology, 10th Military Research Hospital, Bydgoszcz, Poland; ^3^Department of Neurosurgery and Neurology, Faculty of Health Sciences, Ludwik Rydygier Collegium Medicum, Nicolaus Copernicus University, Bydgoszcz, Poland; ^4^Department of Positron Emission Tomography and Molecular Imaging, Ludwik Rydygier Collegium Medicum, Nicolaus Copernicus University, Bydgoszcz, Poland; ^5^Department of Nuclear Medicine, Franciszek Lukaszczyk Oncology Center, Bydgoszcz, Poland; ^6^Department of Oncology and Brachytherapy, Faculty of Medicine, Ludwik Rydygier Collegium Medicum, Nicolaus Copernicus University, Bydgoszcz, Poland; ^7^Department of Neurooncology and Radiosurgery, Franciszek Lukaszczyk Oncology Center, Bydgoszcz, Poland

**Keywords:** biopsy, FET-PET, histopathology, MRI, stereotactic biopsy

## Abstract

Neuroimaging based on O-[2-(18F)fluoroethyl]-l-tyrosine (FET)-PET provides additional information on tumor grade and extent compared with MRI. Dynamic PET for biopsy target selection further improves results but is often clinically impractical. Static FET-PET performed at two time-points may be a good compromise, but data on this approach are limited. The aim of this study was to compare the histology of lesions obtained from two challenging glioma patients with targets selected based on hybrid dual time-point FET-PET/MRI. Five neuronavigated tumor biopsies were performed in two difficult cases of suspected glioma. Lesions with (T1-CE) and without contrast enhancement (T1 and T2-FLAIR) on MRI were selected. Dual time-point FET-PET imaging was performed 5–15 min (PET10) and 45–60 min (PET60) after radionuclide injection. The most informative FET-PET/MRI images were coregistered with MRI in time of biopsy planning. Five biopsy targets (three from high uptake and two from moderate uptake FET areas) thought to represent the most malignant sites and tumor extent were selected. Histopathological findings were compared with FET-PET and MRI images. Increased FET uptake in the area of non-CE locations on MRI correlated well with high-grade gliomas localized as far as 3 cm from T1-CE foci. Selecting a target in the motor cortex based on FET kinetics defined by dual time-point PET resulted in a grade IV diagnosis after previous negative biopsies based on MRI. An additional grade III diagnosis was obtained from an area of glioma infiltration with moderate FET uptake (between 1 and 1.25 SUV). These findings seem to show that dual time-point FET-PET-based biopsies can provide additional and clinically useful information for glioma diagnosis. Selection of targets based on dual time-point images may be useful for determining the most malignant tumor areas and may therefore be useful for resection and radiotherapy planning.

## Introduction

Stereotactic biopsy (SB) of central nervous system (CNS) tumors aims to precisely collect tissue for histopathological examination. SB is often necessary for patients with inoperable gliomas, multifocal CNS tumors, lesions located in deep brain structures, large tumors that are ineligible for resection, suspected lymphomas, and lesions poorly defined on MRI (1). The histopathological diagnosis then forms the basis for oncology decision-making.

SB tries to target locations corresponding to the areas of highest grade, since ultimately this will dictate prognosis and therapy. Routine SB planning is based on MRI analysis, where contrast enhancing (CE) areas are considered the most malignant and are therefore targeted for biopsy. Thus, determining where to target the biopsy in patients with inconclusive MRI images, such as without CE, is difficult. An incorrect diagnosis could limit or even exclude a particular treatment.

SB is a minimally invasive procedure that produces only small tumor samples, so the radiology must maximize the probability of obtaining tissue that delivers the correct histopathological diagnosis. An inadequate or poorly targeted biopsy raises diagnostic concerns, might result in an incorrect diagnosis, or even prevent a diagnosis being made. In the case of ambiguous MRI images, material is often taken from areas that do not necessarily correspond to the most malignant part of the tumor, since ~10–30% of non-enhancing gliomas are actually high grade (2).

Therefore, imaging methods that increase the diagnostic yield are of considerable interest. Multiparametric MRI can help in making the most accurate tumor diagnosis, identifying areas of highest malignancy and a suitable biopsy target (3). Combined MRI and static 18-fluoroethyl-tyrosine (FET)-PET imaging in patients with cerebral gliomas significantly improves the rate of definitive histological diagnosis (4–6). Static FET-PET, which is recommended to start 20 min after tracer injection and usually lasts 20 min, provides high accuracy to determine tumor extent and differentiation of tumor and non-specific uptake in inflammatory areas (7). Furthermore, additional early scanning 5–15 min post-injection may provide additional information in high-grade tumors with early peak uptake (8).

A number of studies have indicated that PET tracer uptake kinetics encode biological information beyond that obtainable from static images, and this data may be helpful for glioma grading (7, 9–11). Therefore, for more accurate tumor imaging, dynamic FET-PET (serial acquisition 0–50 min post-tracer injection) has been recommended (12), and higher grade tumor sites have been found when biopsy targets were based on the results of dynamic PET (13). Dynamic FET-PET, however, requires longer acquisition times, reducing its clinical practicality (14).

Early static FET-PET scans have a higher accuracy for glioma grading than the standard 20-40 min scans (15). Whereas, PET 20–40 min post-injection seems appropriate for delineation of low-grade gliomas as well as IDH-mutant gliomas, high-grade gliomas, and some IDH wildtype gliomas seem to have larger volumes in early images (16). Dual time-point FET-PET is a promising compromise that might improve glioma grading and definition of tumor borders using two static images early (5–15 min post-injection) and late (50–80 min post-injection) after radiotracer injection (17, 18).

Dual time-point FET-PET is more comfortable to the patient than dynamic FET-PET. However, the value of dual time-point FET-PET in SB has yet to be determined. We therefore examined the utility of dual time-point FET-PET for guiding biopsy in two patients with suspected gliomas but inconclusive MRIs.

## Materials and Methods

Both patients gave informed consent at the Nicolaus Copernicus University, Ludwig Rydygier Collegium Medicum, Bydgoszcz, Poland.

### Patient 1

The first patient was a 71-year-old right-handed individual who presented with a single incident of slurred speech that spontaneously resolved. There were no findings on neurological examination. After CT, the patient was initially treated with thrombolysis for presumed ischemic stroke at another center. However, after MRI, a tumor was identified in the deep left hemispheric structures with heterogeneous areas of contrast enhancement. After neurosurgical consultation, the patient was disqualified from resection due to the size of the tumor and its location in the dominant hemisphere, so a stereotactic biopsy was proposed to obtain tissue to guide oncological treatment. The patient was referred for dual time-point FET-PET. At the time of admission, the patient was in good general condition with no neurological deficits and no laboratory abnormalities. The Karnofsky performance status (KPS) was 90.

### Patient 2

The second patient was a 42-year-old right-handed individual who had been diagnosed and treated for glioma since 2004 after presenting with mild partial paraparesis and partial epileptic seizures. The brain MRI revealed extensive tumor in the left hemisphere but without areas of CE. The tumor was polymorphic, hypointense in the T1, and hyperintense in the T2-FLAIR sequence. The tumor was diffuse, multifocal, and had difficulty to define contours. Most parts were located in the motor and premotor cortex of the left hemisphere, but abnormalities were also visible in the deep left hemispheric structures. Due to the tumor extent and location in the dominant hemisphere, resection was not recommended. SB was twice attempted but the results were inconclusive. Initially, a WHO I astrocytoma was diagnosed but this was not consistent with the MRI images. For this reason, another biopsy was performed, again obtaining an ambiguous result of WHO grade I/II glioma. Finally, a diagnosis of gemistocytic astrocytoma, WHO grade II, was made. Both biopsies were planned based on MRI images.

The patient underwent fractionated radiotherapy in 2005. However, over the last 3 years, the frequency of epileptic seizures increased and the hemiplegia worsened. Follow-up MRI scans were inconclusive with no contrast enhancement and no obvious tumor progression. After several oncological and neurosurgical consultations, it was decided to perform SB, suspecting progression of the tumor or perhaps radiation-induced changes. The patient was referred for dual time-point FET-PET. The patient presented for examination with right-sided hemiparesis (grade 3 on the Lovett scale) and mixed aphasia with a predominance of motor aphasia and a slight psychomotor deficiency. The patient had a KPS of 80.

### MRI

Both patients received a standard MRI (axial T2-weighted sequences with a slice thickness of 3 mm and 3D T1-weighted sequences with a slice thickness of 1 mm) before and after administration of gadobutrol [Gadovist™ (EU); Gadavist® (USA)] on 1.5 Tesla scanners (Magnetom Symphony, Siemens; or Signa HDxt, GE Healthcare). The scans were evaluated by experienced neurosurgeons who were not blinded to the results of the 18FET-PET investigation and the histological diagnosis.

### FET-PET and Biopsy Planning

In both patients, two PET images were taken: 5–15 min (PET10) and 45–60 min (PET60) after radionuclide administration. Patients received 250 ± 10 MBq of tracer intravenously. Acquisition was performed with the patient's arms placed alongside the body. A MRI scan was acquired during shallow breathing with the following parameters: CARE Dose 4D, 120 kV, pitch 0.7. The hybrid PET/MRI scan was acquired at 2.7 min per bed position. CT data were used for attenuation correction. Images were reconstructed using a commercial three-dimensional iterative reconstruction algorithm called TrueX+tof (UltraHD-PET; matrix 200 × 200, interval 3 mm, three iterations, 21 subsets). FET tissue uptake was expressed post-biopsy using the standardized uptake value (SUV), dividing the radioactivity (MBq/ml) in the tissue by the radioactivity injected per gram of body weight.

The day before the procedure, both patients underwent MRI examination of the head with T1 with (T1-CE) and without contrast (T1), T2, and T2-FLAIR. PET, MRI, and CT image fusion was performed at the planning station (BrainLAB software, BrainLAB AG, Feldkirchen, Germany) in the operating unit.

Five tumor sites were selected, and tissue was collected by serial SB. In patient 1, tissue was obtained from (i) an area of high FET uptake outside the area of CE; (ii) an area of CE inside the high FET uptake; (iii) at the tumor margin (change of signal at time T2-FLAIR, T1-CE, and at the low of 18-FET uptake in PET10 and moderate in PET60); and in patient 2 from (iv) an area of high FET10 and high FET60 uptake inside high T2-FLAIR signal without CE at time T1; and (v) at the tumor margin at a change in signal at T2-FLAIR in the MRI and at the moderate FET uptake in PET10 (1.2 SUV) and PET60 (1 SUV).

**Figure 1:**
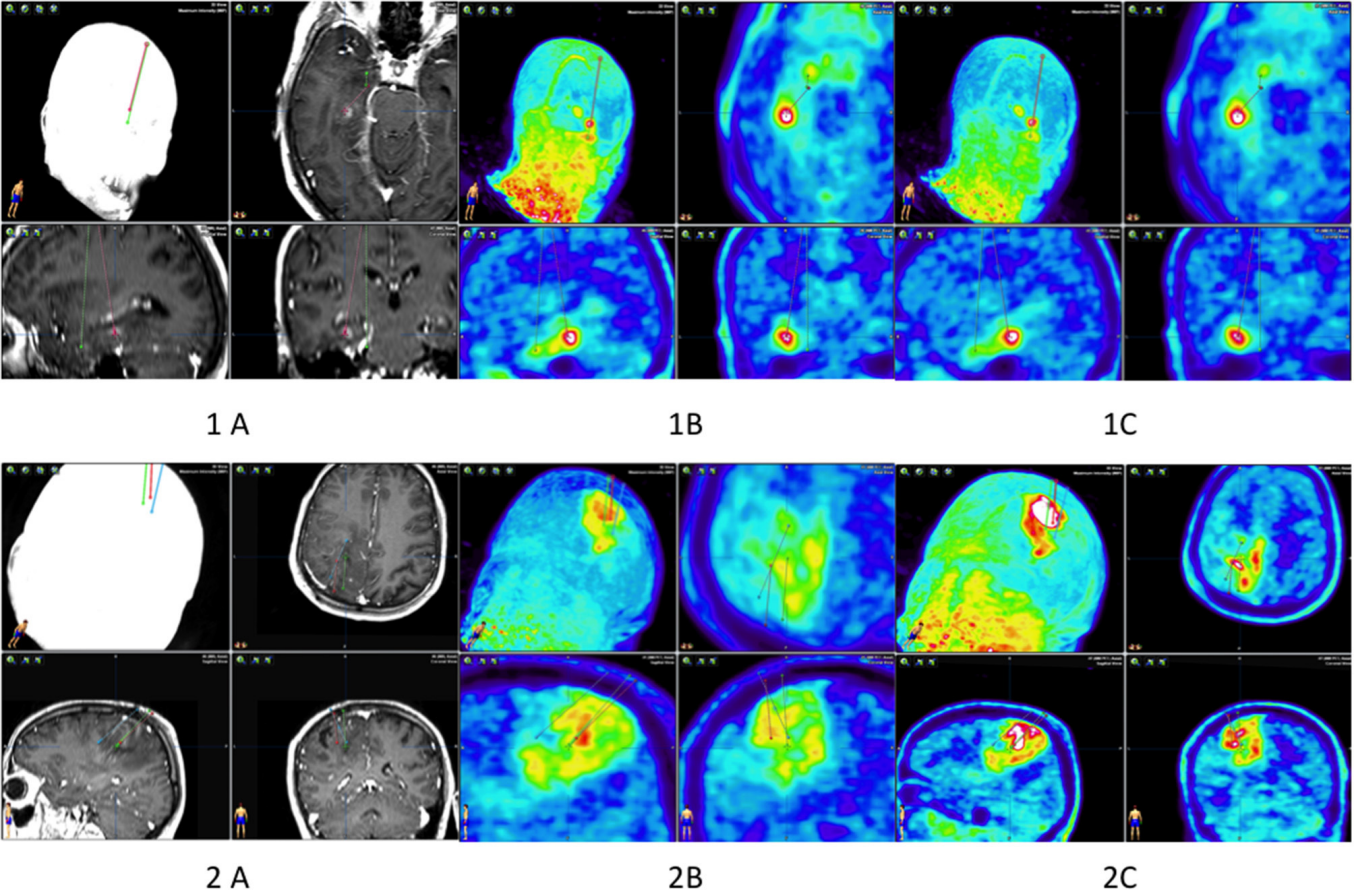
Biopsy planning of patients 1 and 2. Patient 1: two biopsy trajectories in the MRI **(A)**, in PET 10 **(B)**, and in PET 60 **(1C)**. Patient 2: three biopsy trajectories in the MRI **(A)**, in PET 10 **(B)**, and in PET 60 **(2C)**.

In the first patient, collecting material required planning two trajectories with an entry point from one trepanning hole (Figure 1). No complications occurred after the procedure. The patient did not develop any neurological deficits. In the second patient, one trajectory was sufficient to retrieve the material. After the procedure, the patient did not develop any new neurological deficits.

On the first day post-procedure, head CT examination was performed in both patients to exclude CNS bleeding. On the second day, the patients were discharged in the same neurological state as on admission.

### Dual Time-Point FET-PET Data Analysis

A neurosurgeon and nuclear medicine specialist selected the optimal biopsy target based on dual time-point PET/MR images after subjective visual assessment of PET data using the following optical scale: 1, low PET uptake value (SUV < 2×); 2, moderate uptake value (SUV 2–2.5); and 3, high uptake value (> 2.5×) in relation to uptake in the symmetrical brain. Lesions were assessed by maximal SUV in biopsy areas on dedicated viewing software for integrated imaging (Syngo.via; Siemens Healthcare, Erlangen, Germany) by a nuclear medicine specialist and radiation oncologist experienced in PET/CT treatment planning. The ROI in the healthy appearing contralateral brain hemisphere was determined symmetrically to each target point, and the SUVmean in this ROI of normal brain was defined. 18F-FET uptake in the targeted tumor area was determined by a three-dimensional autocontouring process using a tumor-to-brain ratio (TBR) of at least 1.6 surrounding each target point as described by Rapp et al. (19).

### Stereotactic Biopsy

Multimodal imaging-guided stereotactic serial biopsies were performed under local anesthesia using a modification of the Riechert head ring and a workstation for multiplanar trajectory planning (Target@1.19, BrainLAB). Multimodal planning was accomplished after coregistration of CT, MRI (including T1- and T2-weighted sequences), and FET-PET (i-plan stereotaxy, BrainLAB) for better visualization of the tumor, the tumor/vessel interface, and intratumoral metabolic activity; the three-dimensional workstation allowed the simulation of any given trajectory. A biopsy was taken *via* a 7–10 mm skin incision and a 2-mm bore hole along a trajectory representative of that defined on MRI and selected PET images. Using microforceps, the maximum amount of tissue per biopsy specimen was 1 mm^3^. Multiple specimens were taken from selected targets along the chosen trajectory. The neurosurgeon at the time of biopsy was blinded to SUV results. The biopsy targets were performed by visual (qualitative) selection. After biopsy, SUV value was analyzed in iPlan stereotaxy software (Brainlab, Munich Germany) by neurosurgeon and radiation oncologist.

### Histological Evaluation

Intraoperative assessment of the samples was based on the cytopathological method by an experienced neuropathologist. The samples prepared on the slides were stained with methylene blue and evaluated under a microscope. In the post-operative diagnosis, the paraffin method with basic staining with hematoxylin and eosin was used. *MGMT* promoter methylation, loss of 1p and 19q, and *IDH1* and *IDH2* mutation were performed as previously (20).

Tissue obtained by stereotactic biopsy is oligobiopsy material and therefore small, with most samples not exceeding 1 mm in diameter. Depending on the biopsy target, different microscopic appearances may be observed within the same tumor; for example, a different morphology is often observed in the peripheral and central zones of the tumor. Therefore, due to the small tissue volumes, no complete set of histological features characteristic for a given type of cancer was applied here. Nevertheless, anaplastic astrocytoma had a higher density of cells than benign astrocytomas, with pleomorphism and hyperchromasia. Multinuclear cells were visible, and the stroma had a delicate, filamentous structure. Blood vessels were thin walled and not numerous. Glioblastoma multiforme contained dense, pleomorphic cells, necrotic areas with peripheral nuclear palisading, and endothelial proliferation. Cells were variable from small and hyperchromatic and scanty cytoplasm to mono- or multinuclear larger, polygonal, or oval cells. Some nuclei were bizarre, and vessels were thick walled and numerous.

## Results

### Value of Dual Time-Point FET-PET Imaging in Defining High-Grade Glioma Extent and Diagnosis

**Figure 2:**
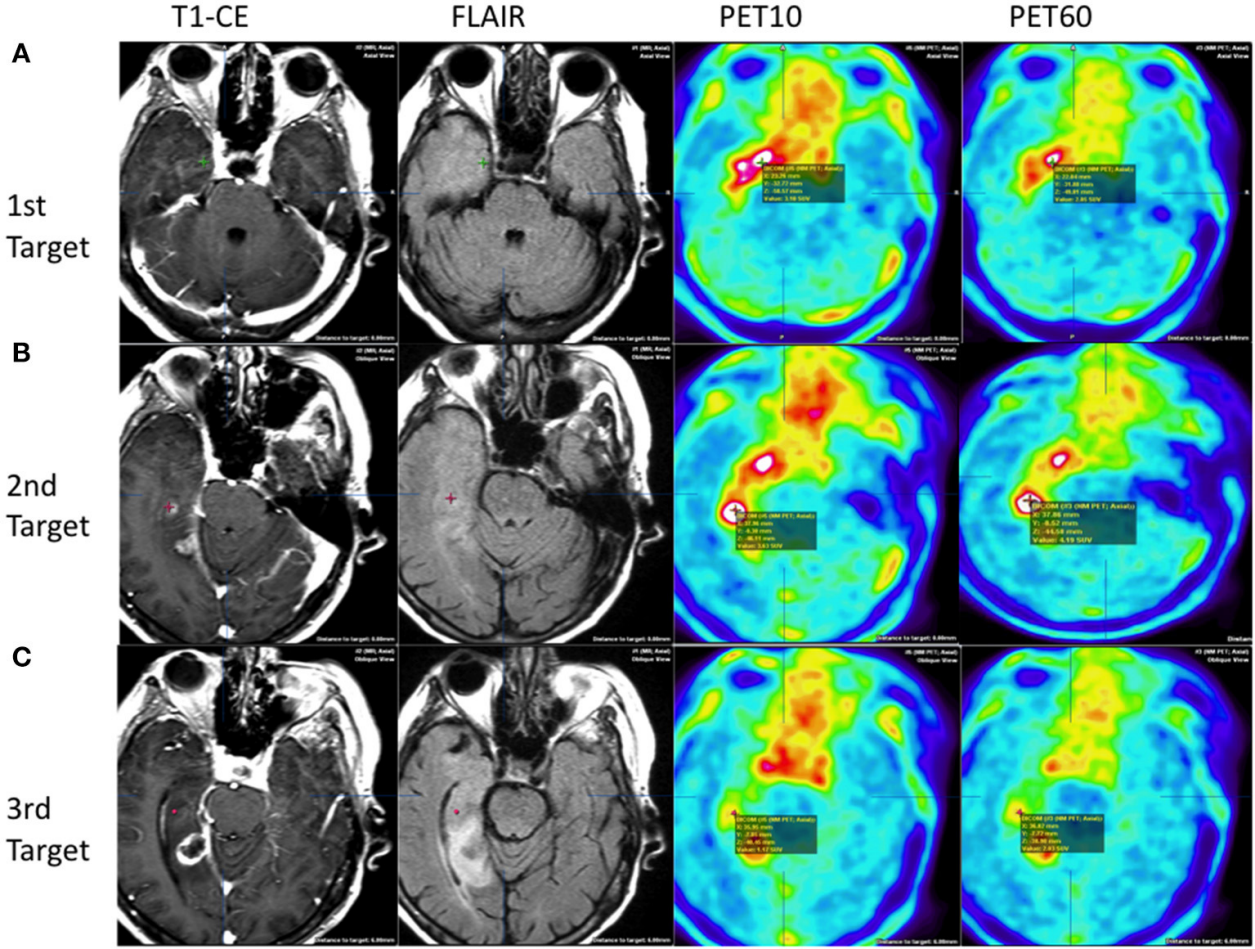
Patient 1: heterogenous tumor biology and uptake behavior. Comparison of three target points in MRI and PET images. Upper row **(A)** first target based on early PET:WHO IV: without contrast enhancement on T1+CE, normo/hyperintensities on FLAIR, high FET uptake of 3.10 at PET10, and high uptake of 2.85 at PET60. Middle row **(B)** second target based on MRI, HP: WHO III: with contrast enhancement on T1+CE, hyperintensities on FLAIR, and high FET uptake of 3.63 at PET10 and 4.19 PET60. Lower row **(C)**: third target based on late PET at the tumor periphery HP: WHO III: without contrast enhancement on T1+CE, normointensities on FLAIR, low FET uptake of 1.17 at PET10, and increased up to 2.03 at PET60.

In patient 1, there were multifocal areas of high FET-PET tracer uptake that were mismatched with the MRI findings. The first focus was located at a considerable distance from the areas of contrast enhancement and T2-FLAIR, and the highest uptake was present in PET10 (Figure 2A) but lower in PET60. The distance between T1-CE and PET10 areas was over 3 cm. In turn, it was not possible to locate an area where CE remained outside the areas of high FET uptake. The second location of highest uptake in PET60 corresponded to the area of contrast enhancement in the MRI (Figure 2B). The histological diagnosis in the first locus present in PET10 was a grade IV malignant glioma, while the histological diagnosis of the second location was a WHO grade III lesion. At the periphery of the tumor in PET60 (Figure 2C), the neuropathologist could not find tumor in the biopsy obtained. As a result, glioblastoma multiforme (GBM) WHO IV was diagnosed overall. The volume of high uptake in PET was greater than the volume of contrast enhancement in MRI, and the tumor margins in PET were more conclusive than those seen in the MRI (Figure 2C).

### Value of Dual Time-Point FET-PET With Regard to Uptake Kinetics

**Figure 3:**
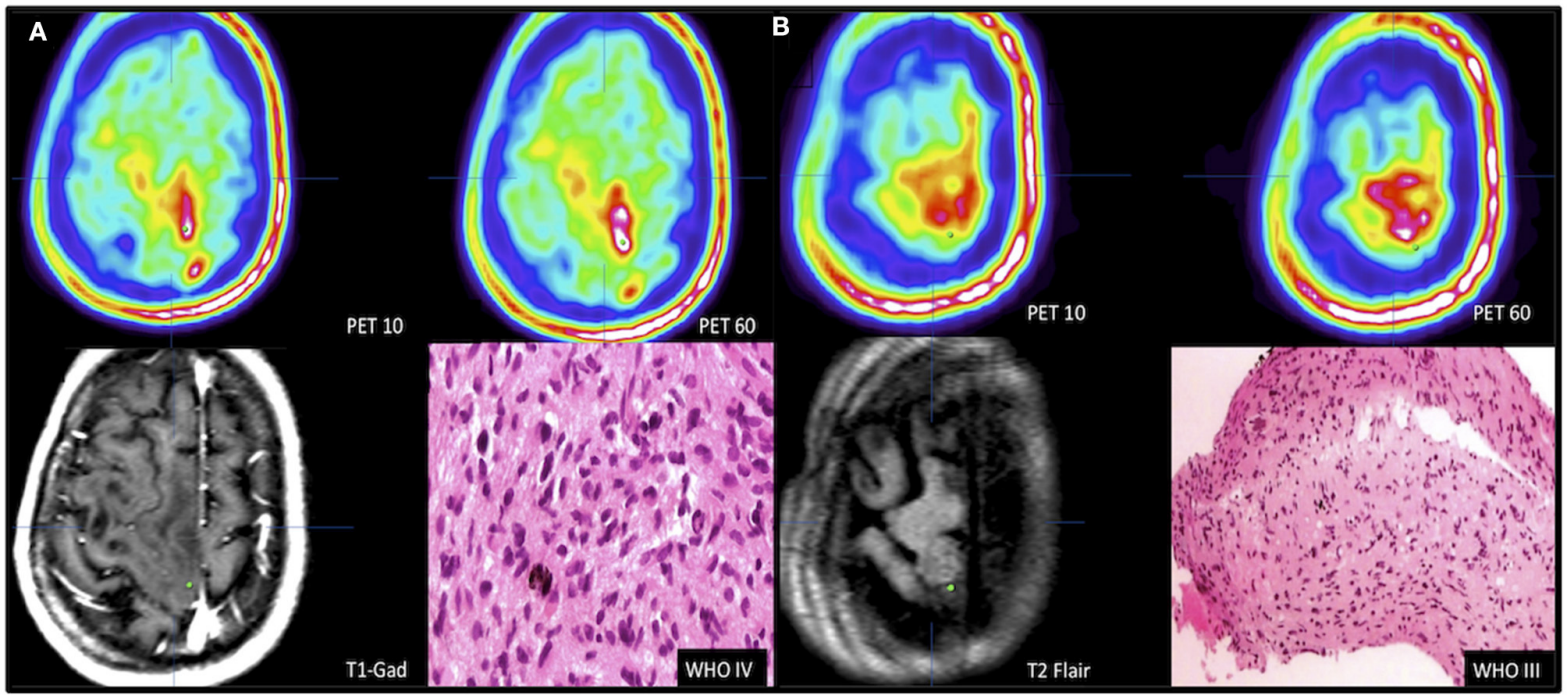
Selection of biopsy target based on dual time point. Patient 2 presented with tumor of eloquent area. Target of highest malignancy **(A)** was selected using FET kinetics defined in dual time-point PET images with no contrast enhancement in the T1-CE sequence. PET10 lesion defined by FET uptake hotspot (1.88 SUV) was expected to be highly malignant (white area); PET60 lesion defined by coexistence of hotspots (white areas) (2.64 SUV). Biopsy target marked by a green dot. Biopsy from hotspot of early occurrence showed GBM. **(B)** Defining the tumor border by targeting the tumor periphery (green dot, PET10 1.26 SUV and PET60 1.1 SUV) in area of moderate FET uptake visible on dual time-point PET with no contrast enhancement in T1+gadolinum sequence and no clear changes visible on T2-FLAIR MRI image. Biopsy result was grade III astrocytoma.

FET uptake kinetics were noted in the second case and were useful for biopsy planning and diagnosis. Part of the tumor was defined by increased FET uptake at PET10, but the highest volume (multifocal) and uptake were noted at PET60 (Figure 3A). Tracer uptake was not increased in the deep left hemispheric structures. Tissue was obtained from the area where FET uptake was visible just after tracer administration; however, this area was located near the motor and premotor cortex. The histological diagnosis from this area of high uptake was a grade IV glioma. The material collected from the area of moderate uptake value in both studies was diagnosed as grade III glioma (Figure 3B). The biopsies, imaging findings, and histological diagnoses are summarized in Table 1.

**Table 1:** Summary of the biopsies, imaging findings, and histological diagnoses.

**Biopsy**	**Patient**	**Location**	**Histopathology result**	**T1 gadolinium contrast enhancement**	**T2 FLAIR**	**Early FET-PET at 5–15 min optical assessment**	**Late FET-PET at 40–60 min optical assessment**	**Early ROI parameters**	**Late ROI parameters**
1	1	Right temporal	WHO IV *MGMT*-methylated *IDH* wildtype without 1p/19q codeletion	Absent	Isointense	High FET uptake	High FET uptake	Background:0.54 SUVmax:3.1	Background: 0.77 SUVmax: 2.85
2	1	Right temporal	WHO III	Present	Hyperintense	High FET uptake	High FET uptake	Background: 0.78 SUVmax:3.63	Background: 0.87 SUVmax: 4.19
3	1	Tumor periphery	WHO III	Absent	Isointense	Low FET uptake	Moderate FET uptake	Background: 0.63 SUVmax: 1.17	Background: 0.77 SUVmax: 2.03
4	2	Motor cortex	WHO IV *MGMT*-methylated *IDH1* mutant without 1p/19q codeletion	Absent	Hypertensive	High FET uptake	High FET uptake	Background: 0.69 SUVmax: 1.88	Background: 0.57 SUVmax: 2.64
5	2	Tumor periphery	WHO III	Absent	Isointense	Moderate FET uptake	Moderate FET uptake	Background: 0.72 SUVmax: 1.26	Background: 0.5 SUVmax: 1.1

*SUV parameters represent tumor area of each target point, background SUV within symmetrical normal brain tissue*.

## Discussion

A well-planned stereotactic biopsy is a procedure that allows for precise material collection and accurate histological diagnosis. Routine biopsy target planning is based on imaging the tumor using MRI T1 sequences with contrast. In general, the most malignant areas of the glioma are in areas of contrast enhancement, where the blood-brain barrier is disrupted. However, biopsies guided by static imaging have shown that MRI imaging does not always accurately correlate with FET-PET images (21, 22). Even more difficult is localizing a biopsy target in non-contrast-enhanced tumors. Selecting the most malignant site using static, single PET imaging is limited (14), and low- and high-grade tumor areas are indistinguishable in static PET images 20–60 min after FET injection (7, 9). Large, single-institution series have shown that biopsy of non-enhancing gliomas based on dynamic PET is feasible and may improve the diagnostic accuracy (13). However, time costs of dynamic PET are high. Early static images improve the accuracy of defining high-grade tumor sites compared with late static images (15).

By adding an early image to the FET-PET protocol, we could select the correct targets in the most malignant parts of lesions suspected to be grade II gliomas by MRI. The dynamic uptake was particularly useful for SB planning in patient 2: material was collected from the area where FET uptake increased in the first few minutes after administration, despite this only representing a small part of the tumor and even though it was located in the motor cortex. Due to previous negative biopsies based on MRI, the decision was made to biopsy this site based on the principle that faster FET uptake should correlate with the most malignant tumor areas, which was confirmed by the subsequent histological diagnosis. In this case, planning a biopsy based on MRI would have most likely resulted in the biopsy of other, less functionally important areas and an incorrect or absent histological diagnosis. An abnormal area in the deep left hemispheric structures on MRI could have been a potential biopsy target, but this area did not show increased uptake on PET so removing material from this area could also have led to an incorrect diagnosis. In this patient, the diagnosis based on PET allowed the subsequent decision to be made to administer chemotherapy.

FET uptake kinetics have been well-described in primary and recurrent gliomas (23), but knowledge of the final histological diagnosis in areas of increased uptake visible on early dynamic or dual time-point images is limited (15), so the value of these approaches is unknown. Our results suggest that areas of high uptake in early images represent malignant tumor, consistent with recent findings (15).

The tumor area is usually larger in FET-PET images than MRI, and high uptake areas do not correlate with areas of contrast enhancement (24), as seen in our first patient. Nowosielski et al. (25) showed that FET uptake was outside the areas of contrast enhancement in 61% of cases and outside hyperintensive areas on T2-FLAIR in 35% of cases. For radiotherapy planning, the congruence of MRI and FET signals for identifying glioma has been shown to be poor (26, 27). However, the nature of high-uptake areas outside MRI areas has not been confirmed histopathologically. In our first patient, it should be emphasized that the area of enhanced FET uptake rather than the area of contrast enhancement was the most malignant area. Moreover, this part of the tumor was invisible in T1-CE MRI images, these images only revealing areas diagnosed as grade III glioma. Standard resections are usually based on T1-CE MRI images. In this case, planning a biopsy solely on the basis of magnetic resonance would probably have given an incorrect histological result, but omitting this area in the resection or planning of radiotherapy might result in disease relapse. Recurrence in such areas has been shown to be evident on MRI up to 10 months after treatment (26).

The use of FET in PET imaging is crucial. Radiopharmaceuticals based on amino acid analogs show low affinity for healthy brain tissue and high affinity for neoplastic tissue (28). Time-activity curves of FET uptake in patients with high-grade gliomas are characterized by an early peak of tracer uptake followed by a steady decrease, whereas patients with low-grade gliomas typically show a steadily increasing pattern (10). Our knowledge of tumor extent in PET is based on a biopsy-controlled study of patients with cerebral gliomas in which a lesion-to-brain ratio (LBR) of 1.6 best separated tumoral from peritumoral tissue in static, single images taken 20–40 min post-injection (4). In addition to standard images taken at 20–40 min post-injection, early images provided complementary information in tumors with early peak uptake (29). It is unknown what value best separates tumoral from peritumoral tissue in these early images. However, the uptake kinetics revealed by dual time-point FET-PET allowed us to observe different shapes and volumes of high uptake areas, similar to Unterrainer et al. (16). We correlated the early and late images taken in dual time-point acquisition with the biopsy results from the center and periphery of the tumors to examine their true extent. In this way, we could identify a tumor periphery (of grade III malignancy) not visible in T2-FLAIR but visible in early and late FET-PET images with moderate FET-PET uptake (above 1 SUV). Although a more comprehensive analysis of a larger cohort of patients is needed to confirm this result, this first experience is promising. Further studies should provide more insights into the threshold FET uptake values between the tumor periphery and normal brain tissue in dual time-point FET-PET. To our knowledge, this is the first biopsy-controlled study taking advantage of FET uptake kinetic to determine not only tumor diagnosis but also tumor periphery characteristics. This may be of value when delineation of tumor extent is needed, such as in radiotherapy planning. A retrospective study of dual time-point FET-PET for radiotherapy planning showed increased uptake in early but not late images that corresponded with sites of recurrence (26).

The surgery of grade II gliomas is based on T2-FLAIR abnormalities, so any tumor not visible on T2-FLAIR is routinely not removed. Moreover, T2-FLAIR has low specificity (4). Different margins and different doses are used in radiotherapy planning depending on the histology (45–54 Gy in grade II and 60 Gy in grades III and IV). Furthermore, temozolomide is routinely used to treat grade IV gliomas. Therefore, the histology of FET-PET abnormalities in areas not visible on MRI is highly predictive for further treatment. Moreover, escalating the radiotherapy dose based on MRI does not improve survival. Therefore, precisely defining the tumor extent is crucial for further advances in glioma radiotherapy. Our first patient had a malignant focus of higher grade (grade IV) glioma over 3 cm from the tumor defined on MRI (grade III). Therefore, the FET-PET biopsy results may alter the radiotherapy and chemotherapy plan in similar cases.

These findings have implications for clinical practice. First, all patients with a lesion suspicious for glioma may benefit from a biopsy based on dual time-point FET-PET acquisition and T1-CE to obtain the most representative tissue to guide future treatment decisions, i.e., the most malignant. Second, the tissue should be taken from the target where increased uptake occurs at an early time-point (5–15 min post-injection), in particular if this uptake decreases at the later time point. Finally, in order to determine the actual extent of glioma infiltration, further research should focus on SB guided by dual time-point FET-PET from sites with moderate uptake to define a threshold between normal and tumor tissue. This could lead to the clinical implementation of this modality in surgery and radiotherapy planning.

In conclusion, while this study represents the preliminary findings from two patients, our observations warrant further study in larger cohorts to precisely assess value of biopsies guided by dual time-point FET-PET imaging. This will help in establishing more accurate diagnosis of gliomas and also improve our knowledge of tumor invasion not visible in MRI or late FET-PET images. FET-PET reveals glial brain tumors more precisely in terms of both volume and histopathological diagnosis and allows for accurate biopsy planning. Selection of targets based on dual time-point images may be useful for determining the most malignant tumor areas and may therefore be useful for resection and radiotherapy planning.

## Data Availability Statement

The raw data supporting the conclusions of this article will be made available by the authors, without undue reservation.

## Ethics Statement

The studies involving human participants were reviewed and approved by Komisja Bioetyczna Collegium Medicum Uniwersytety Mikolaja Kopernika. The patients/participants provided their written informed consent to participate in this study.

## Author Contributions

MH and JF conceived and designed the experiments. JF, BM, JR, and TS performed the experiments. JF, BM, and McH analyzed the data. McH, JF, and JR wrote the paper. All authors contributed to the article and approved the submitted version.

## Conflict of Interest

The authors declare that the research was conducted in the absence of any commercial or financial relationships that could be construed as a potential conflict of interest.
